# Community, Distribution, and Ecological Roles of Estuarine Archaea

**DOI:** 10.3389/fmicb.2020.02060

**Published:** 2020-08-28

**Authors:** Dayu Zou, Hongbin Liu, Meng Li

**Affiliations:** ^1^SZU-HKUST Joint Ph.D. Program in Marine Environmental Science, Shenzhen University, Shenzhen, China; ^2^Shenzhen Key Laboratory of Marine Microbiome Engineering, Institute for Advanced Study, Shenzhen University, Shenzhen, China; ^3^Department of Ocean Science, The Hong Kong University of Science and Technology, Hong Kong, China; ^4^Hong Kong Branch of Southern Marine Science & Engineering Guangdong Laboratory, The Hong Kong University of Science and Technology, Hong Kong, China

**Keywords:** *Thaumarchaeota*, *Bathyarchaeota*, estuarine archaea, distribution, environmental adaptation

## Abstract

Archaea are diverse and ubiquitous prokaryotes present in both extreme and moderate environments. Estuaries, serving as links between the land and ocean, harbor numerous microbes that are relatively highly active because of massive terrigenous input of nutrients. Archaea account for a considerable portion of the estuarine microbial community. They are diverse and play key roles in the estuarine biogeochemical cycles. Ammonia-oxidizing archaea (AOA) are an abundant aquatic archaeal group in estuaries, greatly contributing estuarine ammonia oxidation. *Bathyarchaeota* are abundant in sediments, and they may involve in sedimentary organic matter degradation, acetogenesis, and, potentially, methane metabolism, based on genomics. Other archaeal groups are also commonly detected in estuaries worldwide. They include *Euryarchaeota*, and members of the DPANN and Asgard archaea. Based on biodiversity surveys of the 16S rRNA gene and some functional genes, the distribution and abundance of estuarine archaea are driven by physicochemical factors, such as salinity and oxygen concentration. Currently, increasing amount of genomic information for estuarine archaea is becoming available because of the advances in sequencing technologies, especially for AOA and *Bathyarchaeota*, leading to a better understanding of their functions and environmental adaptations. Here, we summarized the current knowledge on the community composition and major archaeal groups in estuaries, focusing on AOA and *Bathyarchaeota*. We also highlighted the unique genomic features and potential adaptation strategies of estuarine archaea, pointing out major unknowns in the field and scope for future research.

## Introduction

*Archaea* were proposed as the third domain of life by Woese and Fox (1977). The understanding of archaeal distribution, diversity and ecological functions has dramatically changed since then. Originally, archaea were thought to only inhabit extreme environments, and dwell under highly acidic, saline, and high-temperature conditions (Woese et al., 1978). Hence, for decades, they were considered to be obligate extremophiles. However, the discovery of mesophilic archaeal groups in temperate and oxygenated marine waters (DeLong, 1992; Fuhrman et al., 1992) overturned the previous view on the distribution of archaea. Subsequently, their presence was detected in both terrestrial and oceanic waters (DeLong, 1992; Fuhrman et al., 1992; Stein et al., 2002), soils and sediments (Bintrim et al., 1997; Walsh et al., 2005; Biddle et al., 2006; Teske and Sørensen, 2008), and under moderate conditions, which confirmed their ubiquity on a global scale (DeLong and Pace, 2001; Schleper et al., 2005).

The total cell number of bacteria and archaea exceeds 1.2 × 10^30^. These microorganisms mainly inhabit the deep oceanic subsurface (approximately 4 × 10^29^ cells), deep continental subsurface (approximately 3 × 10^29^ cells), soil (approximately 3 × 10^29^ cells) and open ocean (approximately 1 × 10^29^ cells) (Bar-On et al., 2018; Magnabosco et al., 2018; Flemming and Wuertz, 2019). Notably, archaea contribute a considerable proportion to the microbial biomass in moderate ecosystems (Moissl-Eichinger et al., 2018), accounting for approximately 40.0% and 12.8% prokaryotic cells in marginal regions and open ocean sites, respectively (Hoshino and Inagaki, 2019).

Although only few archaeal isolates have been cultured to date, cultivation-independent techniques, including metagenomics and single-cell genomics, have greatly broadened the understanding of archaeal diversity and distribution (Rinke et al., 2013). For a long time, *Crenarchaeota* (mainly consisting of thermophiles) and *Euryarchaeota* (mainly consisting of methanogens and halophiles) were the only defined archaeal phyla.

The number of proposed archaeal phyla has reached nearly 30 in recent years (Adam et al., 2017; Eme et al., 2017; Baker et al., 2020). Accordingly, several archaeal lineages have been proposed as new phyla based on phylogenetic analysis, including *Thaumarchaeota* (Brochier-Armanet et al., 2008), *Korarchaeota* (Barns et al., 1994; Elkins et al., 2008; Reigstad et al., 2010), and *Aigarchaeota* (Nunoura et al., 2011), which led to the establishment of TACK superphylum (Guy and Ettema, 2011) together with *Crenarchaeota*. Recently, metagenomic studies revealed new archaeal phyla belonging to the TACK superphylum, such as *Verstraetearchaeota* (Vanwonterghem et al., 2016), *Geoarchaeota* (Kozubal et al., 2013), *Bathyarchaeota* (Meng et al., 2014; Evans et al., 2015; He et al., 2016), *Geothermarchaeota* (Jungbluth et al., 2017), *Marsarchaeota* (Jay et al., 2018), and *Nezharchaeota* (Wang Y. et al., 2019). The TACK superphylum was proposed as the *Proteoarchaeota* (Petitjean et al., 2014) as the metabolic functions were highly diverse among different phyla. Members of the newly identified Asgard superphylum (i.e., *Lokiarchaeota*, *Odinarchaeota*, *Thorarchaeota*, *Heimdallarchaeota*, *Helarchaeota*, and *Gerdarchaeota*) were suggested to be the closest prokaryotes related to eukaryotes (Spang et al., 2015, 2018; Zaremba-Niedzwiedzka et al., 2017; Seitz et al., 2019; Cai et al., 2020). For example, members of Asgard archaea harbor genes encoding eukaryotic signature proteins previously believed to be specific to eukaryotes (Spang et al., 2015; Zaremba-Niedzwiedzka et al., 2017; Seitz et al., 2019; Cai et al., 2020). Further, the DPANN superphylum comprises *Diapherotrites*, *Parvarchaeota*, *Aenigmarchaeota*, *Nanoarchaeota*, *Nanohaloarchaeota*, and other archaeal groups with extremely small genomes. Some of its members have been hypothesized to have mutualistic or parasitic lifestyles (Baker et al., 2010; Rinke et al., 2013; Castelle et al., 2015; Eme and Doolittle, 2015; Dombrowski et al., 2019). Although most of the archaeal niches and metabolic functions remain unknown, valuable information on archaeal genomics, proteomics, and physiology is available to help us understand the vast diversity and ubiquitous archaea in different environments.

Estuaries act as connectors of the land and ocean, and hence, exhibit unique characteristics that are different from those of terrestrial and oceanic environments (McLusky and Elliott, 2004). Microbial activities in estuaries are stimulated by numerous nutrients carried by a river discharge (Milliman et al., 1985), that may favor the growth of estuarine microbes, including archaea. Previous reports related to estuarine archaea were only focused on limited lineages or genomes. Hence, a systematic research of estuarine archaea may offer a broader view of these microorganisms. Here, we review the community composition and distribution pattern of archaea in global estuarine ecosystems, focusing on the predominant aquatic and sedimentary archaeal groups (i.e., *Thaumarchaeota* and *Bathyarchaeota*, respectively), to better understand the diversity, ecological niches, as well as evolution and adaptation of archaea in estuarine environments.

## Community Structure of Estuarine Archaea

Estuaries, accounting for only 0.4% of the global marine area, are considered to be one of the most productive ecosystems (Longhurst et al., 1995; Cloern et al., 2014) and are important sources of biogenetic emission of methane (Bange et al., 1994; Abril and Borges, 2005). They are also responsible for the delivery of terrigenous silicon, phosphorus, and nitrogen to the ocean, fueling a high primary production in coastal regions (Harrison et al., 2008). In estuaries, the continental freshwater runoff mixes with the coastal seawater. Distinct physicochemical gradients form along the mouth of the estuary as a result of many factors, such as river discharge, monsoons, and anthropogenic interferences (Vieira et al., 2007; Bernhard and Bollmann, 2010). These physicochemical gradients, e.g., decreasing organic compound and nitrogenous nutrient levels, or increasing salinity, and sulfate and chloride levels, may strongly affect the microbial community structure (Alla et al., 2006; Webster et al., 2014). In addition, terrestrial nutrients and microorganisms carried by a river discharge accumulate in estuaries, leading to a relatively high biodiversity and microbial activities therein, and subsequently influencing the biogeochemical and ecological processes of the estuarine ecosystems (Baird et al., 2004; Canfield and Thamdrup, 2009; Webster et al., 2014; Zhou et al., 2017; Liu et al., 2018b). On the other hand, because of the industrialization and urbanization along rivers, large quantities of varied continental inputs have resulted in severe environmental pollution, eutrophication, and oxygen deficit or hypoxia in estuaries (Diaz, 2001; Li et al., 2002; Huang et al., 2003; Howarth et al., 2011; Zheng et al., 2011; Lu et al., 2018). Therefore, a comprehensive understanding of the estuarine microbial community is critical for elucidating their ecological roles in the estuary.

**FIGURE 1:**
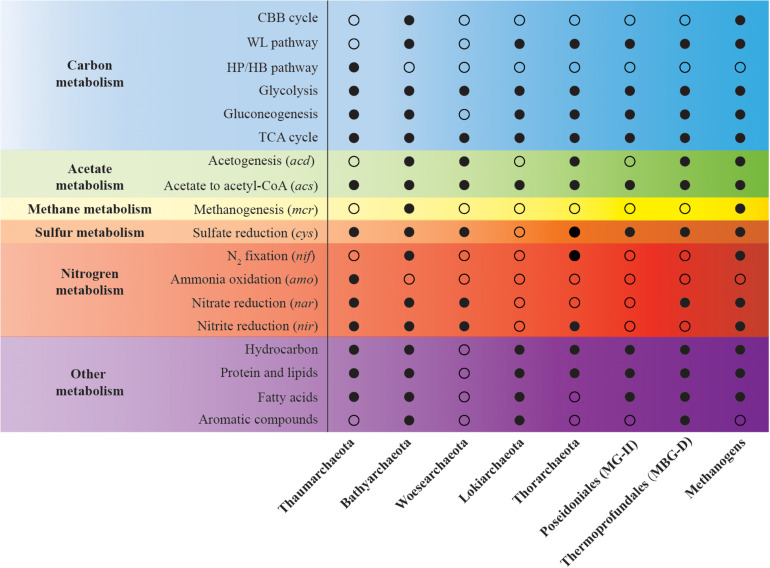
Metabolic potentials of estuarine archaeal groups. Solid circles, metabolic pathways or functional genes identified in some genomes in the group. Empty circles, no reported pathways or functional genes in the genomes in the group. These results are re-summarized mainly based on Baker et al. (2020) and other previous papers, please see the main text and Supplementary Methods for details.

Increasing numbers of archaeal groups are being recognized as vital players in estuarine biogeochemical cycles (Figure 1). These include: *Thaumarchaeota*, which comprise ammonia oxidizing archaea (AOA), one of the key players of ammonia oxidation in estuaries (Francis et al., 2005; Liu L. et al., 2011; Liu Z. et al., 2011; Zou et al., 2019, 2020b); *Bathyarchaeota* (formerly called Miscellaneous Crenarchaeotal Group, MCG), which are hypothesized to be important players in the benthic carbon cycle that, based on genomic analysis, may be able to utilize diverse organic substrates (Meng et al., 2014; Lazar et al., 2015, 2016; He et al., 2016; Zhou et al., 2018a); *Euryarchaeota*, e.g., most methanogens and anaerobic methanotrophs, are responsible for methane production and oxidation in estuaries, respectively (Oremland and Polcin, 1982; Serrano-Silva et al., 2014); and Asgard archaea, such as *Thorarchaeota* and *Lokiarchaeota*, which may participate in some biogeochemical cycles, as suggested by metagenomics (Seitz et al., 2016; Liu Y. et al., 2018; MacLeod et al., 2019; Cai et al., 2020). Recently, Liu et al. (2018b) explored the diversity and community structure of archaea in over 20 estuaries. Analysis of the numbers of operational taxonomic units (OTUs) highlighted the notion that the diversity of archaeal community is higher in low- and middle-latitude estuaries than in high-latitude estuaries. That is probably because the terrestrial and marine archaeal groups are continuously mixed in low- and middle-latitude estuarine regions, affected by high human activity (Xie et al., 2014; Liu et al., 2018b). Further, the archaeal community structure is co-influenced by many environmental factors, such as the latitude, salinity, dissolved oxygen levels, and nutrient conditions (Bernhard et al., 2010; Webster et al., 2014; Xie et al., 2014; Qin et al., 2017; Liu et al., 2018b).

**FIGURE 2:**
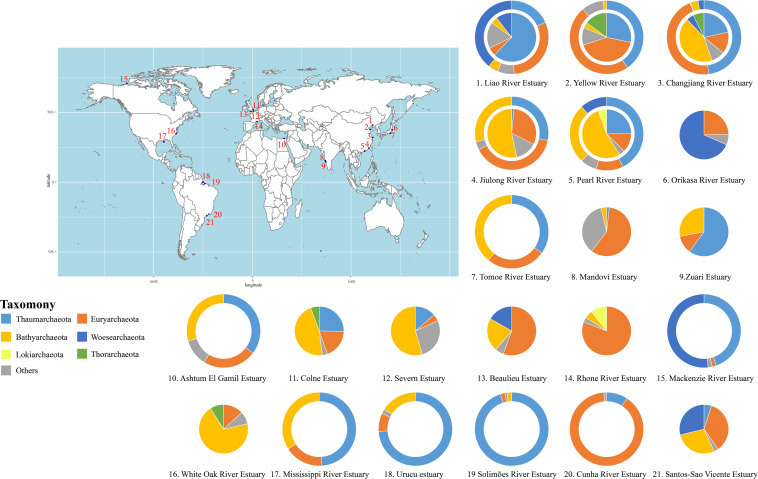
The archaeal community composition (on phylum level) in global estuaries. Outer rings, water samples; inner circles, sediment samples. Please see Supplementary Tables S1, S3 for details.

In this study, we have roughly revealed the archaeal composition in water columns and sediments of different estuaries summarized from published papers (Figure 2 and Supplementary Tables S1, S3 for detailed information). Generally, *Bathyarchaeota* dominate the sedimentary environment in most estuaries, while *Thaumarchaeota* and *Euryarchaeota* are predominant aquatic archaeal phyla. In addition, *Woesearchaeota* account for a high proportion of archaea in both aquatic and sedimentary environments in some estuaries, while *Lokiarchaeota* and *Thorarchaeota* are also observed in some estuaries. As shown in Figure 2, different estuaries harbor different archaeal communities, likely because of specific differences in the geographic location and physicochemical parameters. Liu et al. (2018b) suggested that *Thaumarchaeota* preferentially dwell in low-latitude estuarine environments, while *Bathyarchaeota* and *Euryarchaeota* are more abundant in mid- and high- latitude estuaries, respectively. The response and sensitivity of these archaeal groups to changes in the temperature and nutrient supply are different (Danovaro et al., 2016). This may be responsible for the variations in archaeal community at different latitudes. Intriguingly, although closely located along the west coastline of India, the archaeal communities in the Mandovi Estuary and Zuari Estuary are quite different (Singh et al., 2010; Khandeparker et al., 2017). The authors pointed out that the distinct microbial communities in these two estuaries might be affected by different localized interactions, however, more thorough investigations are needed to explain such differences. Similarly, a recent report revealed seasonal variations of archaeal communities in estuarine and coastal regions (Liu et al., 2020), highlighting the notion that spatiotemporal distribution of archaeal community is co-influenced by environmental parameters, including salinity and temperature. However, other than spatial and temporal variables, technical biases, such as sampling methods, library construction approaches, and different usage of primers and marker genes, might also impact the microbial community composition. Although it is difficult to measure the contribution of all variables, a precise and lucid understanding relies on considering different aspects as much as possible.

## AOA in Estuaries

### AOA Diversity Based on *amoA* Genes

Marine Group I (MG-I) archaea were first detected in the ocean water column (DeLong, 1992). MG-I are ubiquitous in the coastal water, and from shallow marine water to bathypelagic zones, accounting for a considerable fraction of the microbial community in those regions (Karner et al., 2001; Schattenhofer et al., 2009). The ability of archaeal ammonia oxidation was confirmed when the AOA *Nitrosopumilus maritimus* SCM1 (SCM1) was isolated (Könneke et al., 2005). Analyses of the archaeal *amoA* gene encoding the α-subunit of ammonia monooxygenase (AMO) revealed that archaea with an ammonia-oxidizing potential are ubiquitous in natural environments (Francis et al., 2005; Biller et al., 2012; Cao et al., 2013; Cheung et al., 2019). Members of chemolithoautotrophic AOA may be mixotrophic, as they harbor genes encoding extracellular peptidases and carbohydrate-active enzymes (Li et al., 2015; Zou et al., 2019). In addition, because of high affinity for ammonia, low oxygen demand, and tolerance of a wide salinity range, AOA are thought to have more prominent advantages for estuarine ammonia oxidation (Bollmann and Laanbroek, 2002; Martens-Habbena et al., 2009; Mosier et al., 2012b; Qin et al., 2017).

**FIGURE 3:**
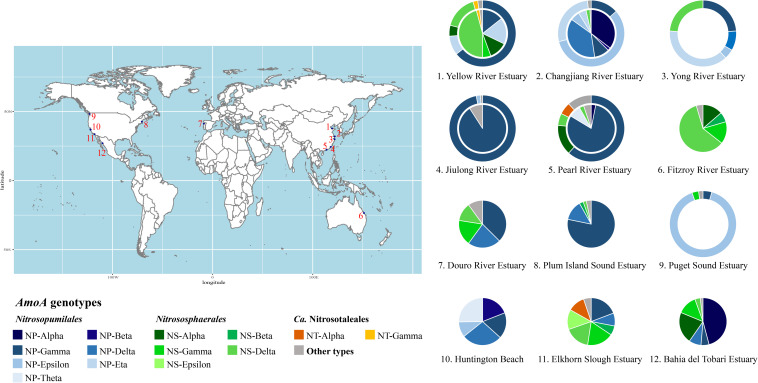
The AOA community composition (based on the *amoA* genotype) in global estuaries. Outer rings, water samples; inner circles, sediment samples. Please see Supplementary Tables S2, S4 for details.

Based on the *amoA* genotypes as suggested by Alves et al. (2018), we summarized AOA community of different estuaries from published papers (Figure 3 and Supplementary Tables S2, S4 for detailed information). In aquatic samples, most estuarine AOA belong to lineage *Nitrosopumilales* (NP), while members of *Nitrososphaerales* (NS) are predominant in sediments, in agreement with previous observations (Alves et al., 2018). The genotypes NP-Gamma (SCM1-like) and NP-Epsilon (WCA) are more abundant in water columns than in sediments. According to Alves et al. (2018), these two groups are distributed in diverse environments, and are enriched in marine and estuarine regions, contributing to approximately 15% and 8% of all *amoA* sequences in global environments, respectively. On the other hand, the NS-Gamma and NS-Delta are more abundant in estuarine sediments, contributing to approximately 13% and 23% of all *amoA* sequences (Alves et al., 2018). The actual community composition varies among different estuaries, emphasizing the point that AOA distribution is environment-specific. Indeed, the approximate abundance of *amoA* genes in estuaries ranges from 1.0 × 10^3^ to 1.7 × 10^8^ copies/L and from 4.9 × 10^3^ to 2.4 × 10^8^ copies/g in water columns and sediments, respectively (Supplementary Table S2). Notably, the technical biases, such as different detection methods, PCR settings and primers, and analysis pipelines among these studies (Supplementary Tables S2, S4), may also have impacts on the results. Hence, they are not directly comparable to each other unless all these issues are carefully considered.

### Distribution Patterns of AOA

Ammonia-oxidizing archaea represent one of the most ubiquitous ammonia oxidizers on Earth, inhabiting both moderate and extreme environments, and also found as putative symbionts of marine invertebrates (Steger et al., 2008; Webster and Taylor, 2012; Erwin et al., 2014). Some AOA cultures grow at temperatures up to over 70°C (de la Torre et al., 2008; Daebeler et al., 2018), and in the range of pH values from 4.0 to 7.5 (Schleper and Nicol, 2010; Stieglmeier et al., 2014). However, the archaeal *amoA* genes have been detected at wider ranges of temperature (as low as 0.2°C and as high as 97°C) (Reigstad et al., 2008), pH (from 2.5 to 9.0), and salinity (from 0 to 38 psu) (Erguder et al., 2009), implying that AOA or *amoA*-encoding archaea (AEA) may be more widespread than current appreciated. Future cultivation or enrichment experiments may offer additional clues to better understand these microorganisms.

According to many *amoA*-based studies, the distribution and abundance of AOA or AEA are strongly associated with the environment. Hence, they can be generally categorized into marine and terrestrial groups (Francis et al., 2005; Biller et al., 2012; Pester et al., 2012; Cao et al., 2013; Yao et al., 2013). The distribution of the marine group is mainly related to the water depth, reflecting the response and adaptation of different ecotypes to different light exposures and ammonia concentrations (Sintes et al., 2013; Luo et al., 2014), while the terrestrial group is mainly partitioned by pH values (Gubry-Rangin et al., 2011).

Based on the archaeal *amoA* genotypes, a community shift of AOA or AEA along the estuary is commonly observed, with salinity one of the most important environmental factors influencing the distribution pattern (Dang et al., 2008; Mosier and Francis, 2008; Santoro et al., 2008; Bernhard et al., 2010; Zhang Y. et al., 2015). Salinity affects the ammonium adsorption in sediments (Boynton and Kemp, 1985), while the correlation between archaeal *amoA* gene abundance and salinity is different in different estuaries. For example, archaeal *amoA* genes are more abundant in low-salinity sediments of the San Francisco Bay than in high salinity regions (Mosier and Francis, 2008), but show positive correlation with salinity in the Elkhorn Slough Estuary (Caffrey et al., 2007). In addition, the abundance and transcriptional activity of archaeal *amoA* genes are high in estuarine regions with medium- and high-salinity (Zhang Y. et al., 2015). However, no significant correlations between the abundance of archaeal *amoA* genes and estuarine salinity were also reported (Santoro et al., 2008; Cao et al., 2011). The distinct seasonal changes of environmental variables in estuaries caused by river discharge may also influence the AOA community (Beman and Francis, 2006; Santoro et al., 2008; Cao et al., 2011).

The abundance and transcription of bacterial *amoA* genes are reduced with decreasing dissolved oxygen (DO), but DO changes do not significantly influence the diversity of archaeal *amoA* genes (Abell et al., 2011). The archaeal *amoA* genes are typically highly abundant in oceanic oxygen-minimum or -deficient zones (Molina et al., 2010; Pitcher et al., 2011; Peng et al., 2013). On the other hand, an increased relative abundance of archaeal *amoA* genes over bacterial *amoA* genes under low DO conditions in some estuaries was also reported (Santoro et al., 2008; Qin et al., 2017). According to a recent study, oxygen availability drives the *Thaumarchaeota* evolution (Ren et al., 2019), and is important for the expansion of AOA habitats from the terrestrial to hadopelagic ecosystems.

Light may also influence the AOA distribution. Archaeal AMO enzymes are more sensitive to photoinhibition than bacterial AMO enzymes (Merbt et al., 2012), which leads to AOA enrichment in the bottom water layers of estuaries (Zou et al., 2019). As another factor, temperature controls the diversity and distribution of AOA in the Westerschelde Estuary (Sahan and Muyzer, 2008). That is because variations in temperature may affect substrate availability, essential for the optimal microbial growth (Andersson et al., 2006). Seasonal variations of light and temperature are important factors controlling the distribution of different AOA species (Liu Q. et al., 2018). AOA are suggested as major players in the nitrogen cycle in low-nutrient environments (Erguder et al., 2009), such as the oligotrophic oceans (Francis et al., 2005). They are also reported as the major ammonia oxidizers in some eutrophic estuaries (Beman and Francis, 2006; Bernhard and Bollmann, 2010; Cao et al., 2011; Jin et al., 2011; Wang et al., 2014; Zou et al., 2019, 2020b) and lakes (Wu et al., 2010; Hou et al., 2013; Zhao et al., 2013; Bollmann et al., 2014). The impact of these environmental parameters, especially the salinity and ammonium concentration, on the distribution of AOA varies with species (Fukushima et al., 2012; Hatzenpichler, 2012; Stahl and de la Torre, 2012), which implies that AOA are well adapted to different environments.

### Genomic Features of Estuarine AOA

Comparative analysis supports the notion that the genomic differences among AOA ecotypes are strongly associated with the habitat. For example, the gene *kefA*, encoding a K^+^ transporter, is rarely present in the terrestrial AOA group and is mainly harbored by marine AOA, which is interpreted as an adaptation to osmotic pressure in aquatic environments (Ren et al., 2019). Further, members of the shallow water group commonly encode the *uvr* system and *pst* systems, which are important for repairing ultraviolet light-induced DNA lesions and phosphate scavenging under phosphorus limitation, respectively; these genes are absent in members of the deep water group, consistent with the physicochemical conditions in deep oceans (Luo et al., 2014; Ren et al., 2019). Oxidative stress is more pronounced in epipelagic water than in deeper water layers, mainly because of a higher abundance of reactive oxygen species generated by many photochemical and photosynthetic processes (Diaz et al., 2013). Consequently, AOA from the epipelagic clade encode more genes related to superoxide dismutase than the bathypelagic clade (Luo et al., 2014). Further, genes related to signal transduction and regulation mechanisms are significantly enriched in the epipelagic clade compared with the mesopelagic clade (Rodriguez-Brito et al., 2006), which may be crucial for their adaptation to the changing upper oceanic environment.

Generally, based on the Cluster of Orthologous Groups (COG) categories, estuarine AOA have a higher proportion of genomic content related to transport and metabolism of amino acids, nucleotides, and lipids than marine AOA (Ngugi et al., 2015). Among many AOA isolates and enrichment cultures, the low-salinity type *Candidatus* Nitrosoarchaeum limnia (*N. limnia*) strain SFB1 enriched from estuarine sediments has several unique genomic features compared with other marine or soil AOA (Blainey et al., 2011; Mosier et al., 2012b). Growth experiments suggest that the *N. limnia* preferentially dwells in low-salinity environments, yet can grow in freshwater and under high-salinity conditions, which presumably is advantageous for adaptation to the estuarine environment, where the salinity typically fluctuates with the seasons (Mosier et al., 2012b). Genomic analysis revealed osmotic adaptation and niche differences between the halotolerant *Nitrosopumilus*-like RSA3 from the Red Sea Basin and other *Nitrosopumilus*-like AOA from epi- and mesopelagic oceans (Ngugi et al., 2015). Similarly, the *N. limnia* genome encodes many mechanosensitive channel proteins, which are necessary to protect microbes from hypoosmotic shock (Blainey et al., 2011). As confirmed by the electron microscopy, *N. limnia* cells are actively motile as they have flagella (Blainey et al., 2011). The genome of *N. limnia*, as well as other estuarine AOA enrichment cultures (strain BG20 and strain BD31) encode genes associated to flagellar biosynthesis and chemotaxis (Blainey et al., 2011; Mosier et al., 2012a).

The physicochemical parameters of estuaries change mainly as a result of river discharge, and it appears that high cell motility of estuarine AOA may be essential for their response to the variations in substrates and oxygen levels. Phosphate usually limits the growth of aquatic microbes in estuarine and coastal regions. Accordingly, estuarine AOA reportedly have many genes related to phosphate transport and regulation systems related to phosphate acquisition (Mosier et al., 2012b; Zou et al., 2019). Alternatively, they may utilize diverse types of phosphorus sources by using polyphosphate enzymes and phosphatases (Qin et al., 2020; Zou et al., 2020a). In eutrophic estuaries, such as the Pearl River Estuary and the Jiulong River Estuary, AOA genomes encode extra genes involved in heavy metal transport and regulation systems, and carbohydrate metabolisms, which may be important strategies to adapt the eutrophication and heavy metal pollution in these estuaries (Zou et al., 2019, 2020a).

Overall, estuarine AOA have evolved prominent coping strategies against osmotic pressure, eutrophication, and potential phosphorus limitation, and can sense and actively seek favorable microenvironments, perhaps via cell motility, thus forming a distinct ecotype in estuaries. However, only few published papers focused on the genomic and physiological features of estuarine AOA. Accordingly, more emphasiss should be placed on depicting their metabolic activity and contribution to nitrification.

### Recent Findings and Research Hotspots Related to *Thaumarchaeota*

The origin and evolution of AOA is one of the research hotspots of recent years. The acquisition of a variant vacuolar-type (V-type) ATPase for AOA, via interphylum horizontal operon transfer, is tightly linked with their habitat expansion to acidic soils and to high-pressure hadopelagic ocean (Wang B. et al., 2019). In addition, the genotypic and gene content variability of marine AOA is driven by phosphorus and ammonia availabilities, and hydrostatic pressure (Qin et al., 2020). The acquisition of high-affinity ammonium transporter (*amt*) and high-affinity *pst* transporter for some marine AOA may play a crucial role in oligotrophic deep oceans (Qin et al., 2020). Therefore, variation in ATPase composition and structure, and genes related to ammonia and phosphorus transport may be important strategies for their adaptation to different environments.

Previous studies suggested that some members of *Thaumarchaeota* are non-AOA and are discovered in diverse environments (Rinke et al., 2013; Weber et al., 2015; Hua et al., 2018; Aylward and Santoro, 2020). Members of non-AOA represent the deepest lineages in the phylogenetic tree of *Thaumarchaeota*, forming the basal group (Group I.1c) (Beam et al., 2014; Hua et al., 2018). Terrestrial non-AOA are thought to be AOA ancestors. The acquisition of the aerobic ammonia oxidation, cobalamin, and biotin biosynthetic pathways for AOA, followed by their habitat expansion to marine environments, may be mainly driven by oxygen (Ren et al., 2019). Members of non-AOA can grow in both anaerobic and aerobic conditions, and oxygen may influence the diversity and structure of non-AOA community (Biggs-Weber et al., 2020). Since only few genomes and enrichment cultures for non-AOA *Thaumarchaeota* are available (Kato et al., 2019), most of their metabolic potentials remain underexplored. Hence, more detailed studies are needed to comprehensively describe their physiology.

Some coastal AOA strains rely on the uptake and assimilation of organic carbon compounds and are considered as obligate mixotrophs (Qin et al., 2014). Although α-keto acids (e.g., pyruvate) enhance the activity of some AOA, an evidence of mixotrophy, α-keto acids also play an important role in H_2_O_2_ detoxification (Kim et al., 2016). Therefore, it is important to clarify whether AOA depend on organic compounds for energy required for growth, or whether these compounds are used in other metabolic pathways. Members of the Group I.1b *Thaumarchaeota* (*Nitrososphaeraceae*) lead a heterotrophic lifestyle in sludge, yet they encode the *amoA* gene (Mußmann et al., 2011). This indicates not all AOA are obligate autotrophic ammonia oxidizers under certain conditions. However, there is no clear evidence that these heterotrophic AOA cannot live autotrophically or are actually mixotrophs. Intriguingly, a group of heterotopic marine *Thaumarchaeota*, a sister group close to AOA rather than affiliated to the basal group, lacks the ability to oxidize ammonia and to engage in AOA-specific carbon-fixation (Aylward and Santoro, 2020; Reji and Francis, 2020). Based on metagenomics data, this group is widespread in marine environments, has smaller genome sizes than AOA, and encode the form III ribulose-bisphosphate carboxylase (RuBisCO) that potentially functions in nucleotide scavenging (Aylward and Santoro, 2020; Reji and Francis, 2020). The discovery of this group altered the knowledge of the metabolic diversity of *Thaumarchaeota*, and established solid connection between the basal non-AOA and AOA groups. Nevertheless, further physiological and biochemical evidence is necessary to confirm these inferred metabolic potentials.

## *Bathyarchaeota* in Estuaries

### Diversity of *Bathyarchaeota* Based on 16S rRNA Genes

Members of *Bathyarchaeota* were first described more than three decades ago (Barns et al., 1996). Thereafter, several close groups have been clustered into a single lineage, the MCG (Inagaki et al., 2003). The new archaeal phylum *Bathyarchaeota* was proposed recently to highlight its deep branching within the tree of Archaea (Meng et al., 2014). Although no pure cultures nor enrichment cultures of its members have yet been obtained, the phylum *Bathyarchaeota* is considered to be a cosmopolitan archaeal lineage with high phylogenetic diversity and abundance (Kubo et al., 2012; Lloyd et al., 2013; Meng et al., 2014; Zhou et al., 2018a). They are found in the terrestrial soil and marine sediments, from pelagic oligotrophic oceans to organic-rich coasts and estuaries (Kubo et al., 2012; Lloyd et al., 2013; Meng et al., 2014; Lazar et al., 2015; Yu et al., 2017; Pan et al., 2019), accounting for a considerable proportion of the microbial community composition in the freshwater and marine sediments (on average, 36 ± 22%) based on the 16S rRNA gene (Fillol et al., 2016). The 16S rRNA gene sequences of the most distant bathyarchaeotal members only share 76% similarity (Fry et al., 2008; Kubo et al., 2012). Consequently, this phylum was categorized into 25 subgroups (Zhou et al., 2018a). Because of river runoff, estuaries harbor many types of terrestrial and marine subgroups of *Bathyarchaeota* (Zhou et al., 2018a). The relatively high concentration of various organic compounds in sediments might be responsible for the high activity and abundance of *Bathyarchaeota* in these ecosystems (Lazar et al., 2016; Xiang et al., 2017).

**FIGURE 4:**
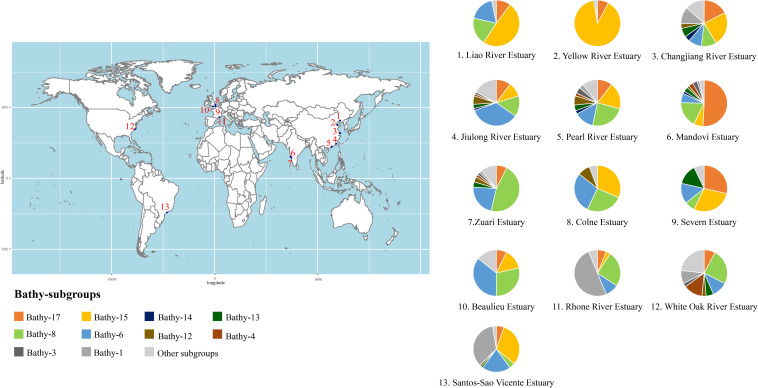
The bathyarchaeotal community composition (based on the 16S rRNA gene sequence) in global estuarine sediments. Please see Supplementary Tables S1, S5 for details.

Below, we discuss in detail the community composition of *Bathyarchaeota* in estuarine sediments based on the archaeal 16S rRNA gene analyses (Figure 4 and Supplementary Tables S1, S5 for detailed information), with the categorization suggested by Zhou et al. (2018a). Although the bathyarchaeotal community varies among estuaries, Bathy-6, -8, -15, and -17 are the predominant subgroups in estuarine sediments, accounting for approximately half of the local bathyarchaeotal community (Figure 4). Other major subgroups identified in estuarine sediments are Bathy-1, -3, -4, -12, -13, and -14, with the remaining community made up of rare subgroups. Xiang et al. (2017) identified several indicator subgroups in estuaries, including Bathy-3, -4, -13, and -16.

### Distribution Patterns of *Bathyarchaeota*

The abundance of *Bathyarchaeota* positively correlates with the concentration of total organic carbon in sediment cores in the South China Sea (Yu et al., 2017) and costal mangroves (Pan et al., 2019, 2020). Enrichment experiments also confirmed that *Bathyarchaeota* (Bathy-8) are able to grow heterotrophically on some organic carbons, such as lignin (Yu et al., 2018). Studies of the relationship between *Bathyarchaeota* and different environmental factors suggest that salinity is one of the most important influential factors (Fillol et al., 2016; Xiang et al., 2017). In one study, bathyarchaeotal community in global sediments was clearly separated into freshwater and saline groups based on principal coordinate analysis, and salinity was the best variable to explain the variance within the bathyarchaeotal community, as confirmed by permutational analysis of variance (Fillol et al., 2016). Further, Fillol et al. (2016) suggested that members of the bathyarchaeotal subgroups are bio-indicator lineages reflecting environments of different salinity (i.e., Bathy-1 and -8 are marine indicators and Bathy-5 and -11 are freshwater indicators), and the evolutionary progression of *Bathyarchaeota* largely occurred in the saline-to-freshwater direction. Because of the lack of pure or enriched cultures, these observations are mainly based on the 16S rRNA genes analysis. Hence, a detailed mechanistic proof of the influence of salinity on *Bathyarchaeota* should be obtained via genomics and, transcriptomic approaches, and in culture-based experiments.

Salinity and other environmental parameters are important for shaping the bathyarchaeotal community in costal and estuarine regions (Liu et al., 2014; Lazar et al., 2015; Yoshimura et al., 2018; Zhou et al., 2018b; Pan et al., 2019; Chen Y. et al., 2020; Zou et al., 2020b). The total abundance of *Bathyarchaeota* increases with the sediment depths in the Pearl River Estuary (Liu et al., 2014), and decreases with the decreasing reductive redox conditions in the sediment in the White Oak River (Lazar et al., 2015), but it remained stable in the surface sediments of the Pearl River Estuary (Zou et al., 2020b) and the Bay of Marennes-Oléron (Hélène et al., 2015). Salinity and ammonium levels are major factors influencing the distribution of *Bathyarchaeota* in surface sediments of the Pearl River Estuary (Zou et al., 2020b). On the other hand, the pH and oxygen levels, rather than salinity, are contributing factors shaping community structure of *Bathyarchaeota* community structure in costal mangroves and lakes (Yoshimura et al., 2018; Pan et al., 2019, 2020). Further, in the southern Yellow Sea and northern East China Sea, water depth, temperature, and salinity are the most influential factors determining the distribution of *Bathyarchaeota* in surface sediments (Chen Y. et al., 2020; Liu et al., 2020). Similarly, water depth is the major variable explaining the partitioned distribution pattern of *Bathyarchaeota* from the shallow marginal sea to the deep northern South China Sea (Zhou et al., 2018b). The prevalence of *Bathyarchaeota* in subsurface environments, and their relatively high abundance in costal and estuarine regions with high nutrient levels, suggests an anaerobic heterotrophic lifestyle and a possible ability to degrade organic compounds.

Although *Bathyarchaeota* are considered to be one of the most persistent and abundant core lineages of the sediment archaeal communities, the distribution of bathyarchaeotal subgroups is thought to be strongly associated with the environment, with a huge diversity within the lineage (Fillol et al., 2016). The *Bathyarchaeota* community is more complex and diverse in mangrove soils and estuary sediments than in other environments (Xiang et al., 2017). Studies suggest that Bathy-6 preferentially dwells in shallow suboxic sediments with low sulfide levels, while Bathy-8 persists in deeper and more reducing marine environments (Lazar et al., 2015; Zhou et al., 2018a). Even so, the predominant subgroup varies with the environment. For example, Bathy-6 predominates in the shallow water sediments of the northern South China Sea (Zhou et al., 2018b), inner surface sediments of the Pear River Estuary (Zou et al., 2020b) and the White Oak River Estuary (Lazar et al., 2015), as well as surface sediments of mangrove wetlands (Pan et al., 2019) and the karstic Lake Cisó (Fillol et al., 2015). Bathy-8 usually dominates in marine sediments and at greater depth (Lazar et al., 2016; Yu et al., 2017), while also dominating in the near-shore surface sediment samples in the northern East China Sea (Chen Y. et al., 2020). Bathy-15 and Bathy-17 are widely detected in freshwater and marine sediments (Fillol et al., 2015; Xiang et al., 2017), and Bathy-15 abundance is relatively stable with changing depth and redox conditions (Hélène et al., 2015; Yu et al., 2017). The environment-specific distribution of these subgroups underlines the distinct metabolic capacities, potential ecological functions and adaptation strategies of *Bathyarchaeota* in specific habitats.

### Ecological Functions and Microbial Interactions of Estuarine *Bathyarchaeota*

Based on genomic analysis, *Bathyarchaeota* are important players in benthic C1 carbon compound cycling, potentially via methane metabolism (Evans et al., 2015) or acetogenesis (He et al., 2016), and they are genetically capable of degrading diverse organic compounds including detrital proteins, polymeric carbohydrates, fatty acids, and aromatic compounds (Meng et al., 2014; Seyler et al., 2014; Evans et al., 2015; Lazar et al., 2016). Their methylotrophic methanogenesis potential was first detected in bathyarchaeotal genome bins from the Surat Basin, which harbor key genes, such as the methyl-coenzyme reductase (MCR) and many methyltransferases, involved in utilizing a wide range of methylated compounds (Evans et al., 2015). Recently, a unique MCR type utilizing butane instead of methane was identified, clustering with the members of *Bathyarchaeota*, indicating a potential for butane oxidation (Laso-Pérez et al., 2016). Metagenomic analysis also revealed differences in metabolic capacities, substrate preferences, and ecological niches of bathyarchaeotal subgroups (Meng et al., 2014; He et al., 2016; Lazar et al., 2016), indicating metabolic flexibility of *Bathyarchaeota*.

Genomic analysis suggests that bin BA1 (Bathy-3) can utilize peptide and glucose and bin BA2 (Bathy-8) can degrade fatty-acids, while the lack of ATP-synthase may indicate the restricted substrate-level phosphorylation for energy (Evans et al., 2015). Bathy-6 genome form the White Oak River Estuarine sediments encodes genes involved in degradation of extracellular plant-derived mono- and polysaccharides, while Bathy-15 genome from the same environment encodes extracellular peptidases, underpinning a heterotrophic lifestyle (Lazar et al., 2016). In addition, bathyarchaeotal genomes (Bathy-1, -6, -15, and -17) from the White Oak River Estuary can generate acetyl-CoA autotrophically from CO_2_ and H_2_ through the Wood–Ljungdahl pathway, which might be involved in acetate generation (Lazar et al., 2016). Further, based on metagenomic and enzymatic analysis in the Guaymas Basin, He et al. (2016) suggested that members of *Bathyarchaeota* (Bathy-13, -16, -21, and -22) are acetogens and can utilize diverse organic substrates for fermentation. Genes related to dissimilatory nitrite reduction to ammonium were also identified in these genomes, suggesting a potential capacity for nitrite reduction (Lazar et al., 2016). Recently, diverse nitrogen metabolism related genes were identified in *Bathyarchaeota* genomes, for example the ammonium transporter (*amt*), hydroxylamine reductase (*hcp*), and nitrogenase iron protein (*nifH*) genes (Pan et al., 2020). Furthermore, potential urea production pathways, including the arginase (*rocF*) and agmatinase (*speB*) pathways, were identified in some subgroups (Bathy-6, -8, and -15) in mangrove sediments (Pan et al., 2020). In addition, some *Bathyarchaeota* may reduce S^0^ to sulfide using the hydrogenase/sulfur reductase (*hydA*), while members of Bathy-15 and -17 encode genes related to sulfate reduction and some Bathy-6 genomes harbor thiosulfate reduction genes (Pan et al., 2020). These findings highlight the important roles of *Bathyarchaeota* in benthic nitrogen and sulfur cycles, and underline their highly diverse metabolism.

Feng et al. (2019) compared genomic capacities of 10 bathyarchaeotal subgroups, and suggested that most *Bathyarchaeota* generally lead a lifestyle relying on both heterotrophic degradation and autotrophic carbon fixation. Recently, members of Bathy-6, -8, and -20 were found to encode potential anaerobic cobalamin biosynthesis pathways (Pan et al., 2020). Further, some *Bathyarchaeota* (Bathy-6) are potentially involved in light-sensing as they have genes for rhodopsin and porphyrin biosynthesis (Pan et al., 2020). In addition, a gene for the Form III RuBisCO and other genes related to the Calvin-Benson-Bassham cycle were identified in members of *Bathyarchaeota* (including Bathy-6, -8, -15, and -17), and their expression was supported by transcript analysis (Pan et al., 2020). This indicates that they might fix CO_2_ via multiple pathways, such as the Wood–Ljungdah pathway and Calvin-Benson-Bassham cycle. Although these findings expand the known metabolic potential of archaea and highlight the pivotal role of *Bathyarchaeota* in benthic carbon cycling, future experiments investigating substrate specificity of these proteins and analyses of the intermediate metabolites will help establish their actual functions.

*Bathyarchaeota* may closely interact with other microbes via the methanogenic and acetogenic processes. For example, other heterotrophic and acetoclastic microbes may feed on acetate generated by *Bathyarchaeota* (He et al., 2016; Lazar et al., 2016), while genomic inference suggests that *Bathyarchaeota* may have an anaerobic methane oxidizing capacity. Possible interactions between *Bathyarchaeota*, sulfate-reducing bacteria and anaerobic methane-oxidizing archaea were proposed (Evans et al., 2015; Zhou et al., 2018a). According to Xiang et al. (2017), *Bathyarchaeota* serve as “keystone species” in diverse environments, maintaining the stability and adaptability of the archaeal community. Potential symbiotic or synergistic relationships between *Bathyarchaeota* and *Thermoprofundales* (marine benthic group-D, MBG-D) (Zhou et al., 2019) were genetically inferred, as they share similar pathways, including acetogenesis and protein-degradation pathways (He et al., 2016; Lazar et al., 2016). However, most of these hypotheses are drawn based on limited genomic information. The detailed metabolic functions and interactions need further physiological exploration using more precise and rigorous experiments.

In summary, although no pure cultured strains of *Bathyarchaeota* have been established to date, the current knowledge of this diverse archaeal phylum in terms of their distribution patterns and metabolic functions is expanding, implying the significance of *Bathyarchaeota* in global biogeochemical cycling.

### Further Research Prospects Related to *Bathyarchaeota*

*Bathyarchaeota* are highly diverse. Generally, each subgroup falls into a family or order level based on the 16S rRNA genes (Yarza et al., 2014), while the classification of subgroups varies in different studies. Phylogenetic trees based on 16S rRNA gene sequence and ribosomal proteins inferred from the available genomes of *Bathyarchaeota* from all subgroups share similar topology (Zhou et al., 2018a). This suggests that the current systematic nomenclature and classification of the 25 *Bathyarchaeota* subgroups is clear and definite. However, few 16S rRNA gene sequences remain ungrouped in the phylogenetic tree (Zhou et al., 2018a), making it difficult to predict their metabolic potentials, as representative genomes are lacking. Accordingly, the diversity of *Bathyarchaeota* deserves further exploration. Feng et al. (2019) reported that the deep-rooted Bathy-21 and -22 subgroups from hydrothermal environments might represent ancient types of *Bathyarchaeota*. In a recent study, several *Bathyarchaeota* genomes were retrieved from sediments of the Costa Rica margin subseafloor by metagenomics; they form a novel lineage in the phylogenetic tree, with suggestions that this lineage may couple methylotrophy to acetogenesis via the Wood–Ljungdahl pathway (Farag et al., 2020). We can expect a more comprehensive understanding of the diversity and evolution of *Bathyarchaeota* with the advances in sequencing technology and analysis approaches.

Previous studies also suggested that members of *Bathyarchaeota* grow on lignin (Yu et al., 2018), which offers valuable information for the enrichment of *Bathyarchaeota*. Similarly, the relative abundance of *Bathyarchaeota* increases upon the addition of humic acid and fulvic acid to paddy soils (Yi et al., 2019). Further, abundance of bathyarchaeotal 16S rRNA genes increases over 30-day incubation both, in biofilms supplemented with humic acids and in sediments supplemented with tryptophan (Compte-Port et al., 2020). The knowledge of the physiology, metabolic capacities, and adaptive strategies for *Bathyarchaeota* mainly based on genomic inference and is still rudimentary. Hence, successful enrichments or pure cultures, with the ensuing physiology and biochemistry experiments would accelerate research into these mysterious archaea, confirm the inferred genomic features, and allow improved understanding of their diversity and adaptation.

## Other Active Archaeal Groups Inhabited in Estuaries

Although their abundance is relatively lower than that of the dominant *Thaumarchaeota* and *Bathyarchaeota*, other archaeal groups that inhabit in the estuaries may be also crucial players in the estuarine biochemical cycles, such as the methanogens (Jiang et al., 2011; Hu et al., 2016; Chen S. et al., 2020), Marine Group II (MGII) (Massana et al., 2000; Liu et al., 2014; Zhang C. L. et al., 2015; Xie et al., 2018), *Thermoprofundales* (Biddle et al., 2006; Lloyd et al., 2013; Zhou et al., 2019), and Asgard (Zaremba-Niedzwiedzka et al., 2017; MacLeod et al., 2019) and DPANN archaea (Rinke et al., 2013; Castelle et al., 2015).

### Involvement of Diverse *Euryarchaeota* in Different Biogeochemical Cycles

The presence of MGII archaea in marine water was first reported over two decades ago. They are important planktonic archaea present in surface waters of both pelagic oceans, and in the coastal or estuarine environments, from the tropics to polar regions (Massana et al., 2000; Zhang C. L. et al., 2015). Currently, MGII archaea are proposed to be an order-level lineage namely, *Candidatus* Poseidoniales in the phylum *Euryarchaeota*, with 21 genera affiliated to two families, *Candidatus* Poseidonaceae fam. nov. (formerly subgroup MGIIa), and *Candidatus* Thalassarchaeaceae fam. nov. (formerly subgroup MGIIb) (Rinke et al., 2019). Genome inferred metabolic potential suggests a photoheterotrophic lifestyle for most MGII, and the capacity to degrade proteins, lipids, and other organic compounds, with varied cell motility among different genera (Rinke et al., 2019). The wide distribution of this photoheterotrophic archaeal plankton indicates their important role in carbon cycling, especially in surface waters.

Methanogenesis accounts for a large portion of global methane emission. This process is mainly performed by diverse methanogenic archaea utilizing H_2_/CO_2_, methyl compounds, or acetate to anaerobically generate methane (Thauer et al., 2008). Early studies suggested that methanogens belong to six *Euryarchaeota* genera, as class I methanogens (*Methanococcales*, *Methanopyrales*, and *Methanobacteriales*) and class II methanogens (*Methanosarcinales*, *Methanomicrobiales*, and *Methanocellales*) (Bapteste et al., 2005; Adam et al., 2017). Recently, several new methanogenic archaeal groups within *Euryarchaeota* were identified based on the *mcrA* gene (encoding α-subunit of the methyl-coenzyme M reductase), including *Methanomassiliicoccales* (formerly RC-III) (Borrel et al., 2014), *Methanofastidiosa* (formerly WSA2) (Nobu et al., 2016) and *Methanonatronarchaeia* (Sorokin et al., 2017). Further, archaeal *mcrA* genes have been identified in different archaeal phyla, such as *Bathyarchaeota* (Evans et al., 2015), *Verstraetearchaeota* (Vanwonterghem et al., 2016), *Geoarchaeota* (Wang Y. et al., 2019), and Nezhaarchaeota (Wang Y. et al., 2019), indicating a vast diversity of potential methanogenic archaea.

Methanogenic communities have been described in diverse soil and sedimentary habitats, such as paddies, wetlands, lakes, estuaries, and geothermal or hydrothermal environments (Thauer et al., 2008; Serrano-Silva et al., 2014; Wen et al., 2017). The structure and distribution of methanogens are driven by a series of physicochemical parameters in estuaries. For example, pH strongly influenced the activity of methanogens that use acetate or H_2_ (Kim et al., 2004; Kotsyurbenko et al., 2007), and increasing salinity reportedly to inhibits hydrogenotrophic methanogens, while enhancing acetoclastic methanogenesis (Liu et al., 2016). Hence, the community structure of estuarine methanogens usually exhibits seasonal or spatial variation (Chen S. et al., 2020; Zhang et al., 2020). Among the different habitats, the highest richness of methanogenic linages is observed in estuary sediments (Wen et al., 2017), suggesting higher diversity of methanogens in estuaries than in other habitats.

In addition to *Bathyarchaeota*, *Thermoprofundales* (formally MBG-D archaea) is another important sedimentary archaeal group ubiquitously distributed in marine subsurface ecosystems, and considerably contributing to the benthic biogeochemical cycles (Biddle et al., 2006; Lloyd et al., 2013). According to their unique phylogenetic position and metabolic potentials, MBG-D archaea were recently proposed as a new order *Thermoprofundales* within the class *Thermoplasmata* in the phylum *Euryarchaeota*, with 16 subgroups (Zhou et al., 2019). MBG-D archaea are most abundant in marine sediments and coastal regions, such as mangroves and estuaries, and subgroup distribution is strongly associated with specific environments, illustrating niche-specific adaptation (Zhou et al., 2019). Metagenomics provide insight into the metabolic potential and ecological functions of MBG-D archaea. Such potential includes exogenous protein mineralization, acetate and ethanol through generation via fermentation, and autotrophic growth linked to the Wood–Ljungdahl pathway (Lloyd et al., 2013; Lazar et al., 2017; Zhou et al., 2019). Microbial co-occurrence analyses indicate close interactions of MBG-D archaea with *Bathyarchaeota*, *Lokiarchaeota*, and anaerobic methanotrophic archaea in diverse environments (Zhou et al., 2019), suggesting potential synergistic or syntrophic relationships between these archaeal groups, and highlighting the important role of MBG-D archaea in benthic ecosystems.

### The Ubiquitous and Diverse Asgard Archaea

Asgard archaea are considered as the bridge between eukaryotes and prokaryotes because they are phylogenetically close to eukaryotic cells and encode many genes related to eukaryotic signature proteins (Zaremba-Niedzwiedzka et al., 2017). Asgard archaea have been commonly described in a wide range of habitats, from freshwater to marine environments, such as anaerobic marine, estuarine and limnic sediments, and pelagic waters (MacLeod et al., 2019). They are also more abundant in methane-rich or hydrothermal environments than in terrestrial soil or freshwater environments Among Asgard archaea, *Lokiarchaeota* and *Thorarchaeota* are observed in diverse environments (Zaremba-Niedzwiedzka et al., 2017; Liu Y. et al., 2018; MacLeod et al., 2019; Cai et al., 2020), *Odinarchaeota* are most abundant in geothermal environments, and *Heimdallarchaeota* are enriched in marine sediments. Genomic explorations reveal that Asgard archaea might be important players in the nitrogen and sulfur cycles (MacLeod et al., 2019). Different lineages have diverse metabolic potentials. For example, *Lokiarchaeota* are proposed to utilize halogenated organic matter and are hydrogen-dependent (Sousa et al., 2016; Manoharan et al., 2019); *Thorarchaeota* are thought to be mixotrophy and may be capable of acetogenesis (Seitz et al., 2016; Liu Y. et al., 2018); *Heimdallarchaeota* have the potential to sense light (Pushkarev et al., 2018; Bulzu et al., 2019); *Helarchaeota* possibly oxidize hydrocarbon in anaerobic environments (Seitz et al., 2019); and *Gerdarchaeota* may use both organic and inorganic carbon (Cai et al., 2020). Furthermore, Asgard archaea are proposed to play key roles in diverse environments via microbe−microbe interactions, such as signaling, metabolite exchange, and other biotic or physicochemical activities (MacLeod et al., 2019; Imachi et al., 2020).

### The Ubiquitous and Diverse DPANN Archaea

The discovery of the ultra-small ecto-symbiotic archaeon *Nanoarchaeum equitans* has changed our understanding of archaea. It is understood to be a parasitic ectosymbiont of *Ignicoccus* and other *Crenarchaeota*, and belongs to a separate new archaeal phylum *Nanoarchaeota* (Huber et al., 2002; Waters et al., 2003). Additional lineages closely related to *Nanoarchaeota* have since been identified via single-cell and metagenomic approaches. This led to the recognition of the superphylum DPANN, as most of these microbes have small genomes and limited metabolic capacity (Rinke et al., 2013; Castelle et al., 2015).

*Woesearchaeota* (formerly Deep-sea Hydrothermal Vent Euryarchaeota Group 6, DHVEG-6) are one of the most ubiquitous and abundant archaeal phyla in the superphylum DPANN (Teske and Sørensen, 2008; Castelle et al., 2015). Their habitats span terrestrial waters and soils (Castelle et al., 2015), costal and estuarine regions (Long et al., 2016; Liu et al., 2018b), marine environments (Ding et al., 2017), and even the human microbiome (Koskinen et al., 2017). Based on phylogenetic analysis, Liu et al. (2018a) proposed 26 subgroups for this highly diverse phylum, and suggested oxygen might as a significant factor driving the distribution and evolution of *Woesearchaeota*. Low- and mid-latitude estuaries harbor a higher diversity and abundance of *Woesearchaeota* than high-latitude estuaries, and the community structure is mainly partitioned by oxygen, salinity, and temperature (Liu et al., 2018a). An anaerobic syntrophic lifestyle with obvious metabolic deficiencies has been suggested for members of *Woesearchaeota*, highlighting the requirement for metabolic complementarity with other microbes (Castelle et al., 2015; Liu et al., 2018a). Genomic characteristics suggest that *Woesearchaeota* may be able to generate acetate and hydrogen (Liu et al., 2018a). Network and metabolic modeling imply a possible syntrophic relationship between *Woesearchaeota* and some methanogens. For example, *Woesearchaeota* might support the growth of some H_2_/CO_2_-using and acetate-using methanogens to compete with hydrogenotrophic and acetotrophic methanogens, in exchange receiving amino-acids and other compounds (Liu et al., 2018a). Deciphering the functional importance and ecological role of the vastly diverse DPANN archaea is crucial for improved understanding of the evolution of symbiosis in the archaeal domain of life.

## Summary and Perspectives

Archaea are currently understood to be ubiquitously distributed in diverse environments, and are recognized as important players in the global biogeochemical cycles. Advances in culture-independent technologies facilitate the research into most archaeal groups, which expands our understanding of their diversity, distribution, metabolic potentials, and ecological niches. Estuaries usually harbor a highly diverse active microbial community, largely because of the mixing of terrestrial and oceanic species via river runoff. The community composition and distribution patterns of archaea in different estuaries are widely studied. *Thaumarchaeota*, *Bathyarchaeota*, and *Euryarchaeota* are the most abundant estuarine archaeal phyla. Among them, *Thaumarchaeota* and *Bathyarchaeota* are more abundant in water columns and sediments, respectively, while *Euryarchaeota* inhabit both aquatic and sedimentary environments. Many environmental factors drive the archaeal distribution in estuaries, such as geographic location, salinity, and oxygen levels, while the response to these factors varies among the different archaeal groups. Although the knowledge of estuarine archaea is increasing, most studies are mainly based on the analysis of archaeal 16S rRNA gene or other functional genes. Hence, a more detailed view of the metabolic features and environmental adaptations of estuarine archaea is needed. The future research focus may include:

(1) Detailing genomic characteristics of estuarine archaea using metagenomic approaches and single cell sequencing technologies to better delineate their adaptation strategies.
(2) Dissecting the relationship and dependency between certain types of estuarine archaea and environmental factors in enrichment experiments.
(3) Conducting modeling and long-term monitoring analyses to predict and verify the influence of seasonal changes on the estuarine archaeal community.
(4) Determining the ecological importance of archaea in estuaries using various geochemical approaches to quantitatively analyze the roles of archaea in biogeochemical cycling.
(5) Cultivating and isolating estuarine archaea to verify their inferred metabolic capacities, thus providing more accurate and detailed understanding for their ecological niche.

## Author Contributions

DZ and ML conceived the study. DZ wrote the manuscript with the help from all co-authors. All authors contributed to the article and approved the submitted version.

## Conflict of Interest

The authors declare that the research was conducted in the absence of any commercial or financial relationships that could be construed as a potential conflict of interest.
