# Supplementary material for: Community, Distribution, and Ecological Roles of Estuarine Archaea
Source: Front Microbiol. 2020 Aug 28;11:2060. doi: 10.3389/fmicb.2020.02060 (PMC7484942; doi:10.3389/fmicb.2020.02060)
Supplement: Supplementary file 1 [file Table_1.pdf]

Supplementary information

The Community, Distribution and Ecological Functions of Archaea in Estuaries

Dayu Zou[1,2,3], Hongbin Liu[3,4], Meng Li[1,2 *]

[1]SZU-HKUST Joint PhD Program in Marine Environmental Science, Shenzhen University, Shenzhen, China

[2]Shenzhen Key Laboratory of Marine Microbiome Engineering, Institute for Advanced Study, Shenzhen University, Shenzhen, China

[3]Department of Ocean Science, The Hong Kong University of Science and Technology, Hong Kong SAR, China

[4]Hong Kong Branch of Southern Marine Science & Engineering Guangdong Laboratory, The Hong Kong University of Science and Technology, Hong Kong SAR, China

Supplementary methods

The summary of metabolic potentials of different archaeal groups in the Figure 1 were mainly based on previous studies, including Baker et al. (2020) (for *Thaumarchaeota*, *Lokiarchaeota*, and *Thorarchaeota*), Zhou et al. (2018) (for *Bathyarchaeota*), Liu et al. (2018a) (for *Woesearchaeota*), and Zhou et al. (2019) and Zhou et al. (2020) (for MBG-D, MGII and methanogens).

The archaeal community composition for all sites in the Figure 2 were direct retrieved from its original paper without any analysis. Detailed information for sequencing information for total archaea and *Bathyarchaeota* in water and sediment samples (Figure 2 and Figure 4), and archaeal *amoA* genes (Figure 3) can be found in Table S1 and Table S2, respectively.

For the identification of archaeal *amoA* genotypes in each site, all cloned sequences were blast against the database constructed by Alves et al. (2018) using BLASTN, with e-value as 1e-5. For the classification of bathyarchaeotal subgroups for those sites using clone libraries, cloned sequences were blast against the database constructed by Zhou et al. (2018) using BLASTN, with e-value as 1e-5, while the composition for *Bathyarchaeota* in other sites (using high-throughput sequencing) was direct retrieved from the original paper.

The detailed community composition for total archaea (Figure 2), *amoA* genes (Figure 3) and subgroups of *Bathyarchaeota* (Figure 4) were listed in Table S3-35, respectively.

Supplementary tables

Table S1 Detailed sequencing information in the Figure 1 and Figure 3.

Location	Method	Primer pair	Sequences*	OTUs*	Reference
**Water samples**					
Liaohe River estuary	High-throughput sequencing	344F/915R	38179	528	Li et al., 2020
Yellow River estuary	High-throughput sequencing	344F/915R	23216	497	Wei et al., 2016
Changjiang River estuary	High-throughput sequencing	515F/806R	-	-	Sun et al., unpublished
Jiulong River estuary	High-throughput sequencing	958F/1048R	30000	447	Hu et al., 2015
Pearl River estuary	High-throughput sequencing	341F/806R	19861	576	Xie et al., 2018
Tomoe estuary	Clone library	515F/806R	-	22	Liu et al., 2018b
Ashtum El Gamil estuary	Clone library	515F/806R	-	17	Liu et al., 2018b
Mackenzie River estuary	Clone library	109F/934R	144	57	Galand et al., 2008
Mississippi River estuary	Clone library	515F/806R	-	41	Liu et al., 2018b
Urucu estuary	Clone library	21F/958R	143	-	Toyama et al., 2017
Solimões River estuary	Clone library	21F/959R	163	-	Toyama et al., 2017
Cunha estuary	Clone library	515F/806R	-	86	Liu et al., 2018b
**Sediment samples (including Bathyarchaeota)**					
Liaohe River estuary	High-throughput sequencing	344F/915R	12630	891	Liu et al., 2020
Yellow River estuary	High-throughput sequencing	344F/915R	23216	887	Wei et al., 2016
Changjiang River estuary	High-throughput sequencing	344F/915R	12630	520	Liu et al., 2020

Jiulong River estuary	High-throughput sequencing	958F/1048R	30000	507	Hu et al., 2015
Pearl River estuary	High-throughput sequencing	Arch524F/Arch958R	51311	789	Zou et al., 2020a
Orikasa River estuary	Clone library	AN341If/A1391r	-	78	Kaku et al., 2005
Mandovi estuary	High-throughput sequencing	A-967F/B-1046R	14,271	-	Khandeparker et al., 2017
Zuari estuary	Clone library	21F/958R	254	48	Singh et al., 2010
Colne estuary	High-throughput sequencing	109F/958R	11288	224	Webster et al., 2015
Severn estuary	Clone library	SAfGC-PARCH519R	252	45	Webster et al., 2010
Beaulieu estuary	Clone library	109F/915R	-	18	Liu et al., 2018b
Rhone River estuary	Clone library	22F/1391R	-	48	Liu et al., 2018b
White Oak River estuary	Clone library	A24F/Arch915R	964	-	Lazar et al., 2015
Santos-Sao Vicente estuary	Clone library	515F/806R	-	243	Liu et al., 2018b

*The sequence and OTU number for the high-throughput sequencing stand for the normalized number for all samples in the original paper; for the clone library, it stands for the total number for all samples in the original.

-: Data not mentioned in the original paper.

Table S2 Detailed sequencing information in the Figure 2 and general abundance for archaeal *amoA* genes

Location	Primer pair	Clones	OTUs	Abundance	Reference
**Water samples (copies/L)**					
Pearl River estuary	Arch-amoAF/Arch-amoAR	409	104	6.06e6-2.41e7	Li et al., 2013

Yellow River estuary	Arch-amoAF/Arch-amoAR	171	53	6.53e4-1.06e6	Li et al., 2018b
Changjiang River estuary	Arch-amoAF/Arch-amoAR	386	38	6.66e6-1.72e8	Zhang et al., 2014
Jiulong River estuary	CrenamoA23f/CrenamoA616r	-	-	4.69e5-1.81e8	Zou et al., 2020b
Yong River estuary	Arch-amoAF/Arch-amoAR	20	-	2.31e6-6.57e7	Zhang et al., 2015
Puget Sound estuary	Arch-amoAF/Arch-amoAR	143	16	1.00e3-2.10e6	Urakawa et al., 2014
**Sediment samples (copies/g)**					
Pearl River estuary	Arch-amoAF/Arch-amoAR	145	36	9.60e6-5.10e7	Jin et al., 2011
Yellow River estuary	Arch-amoAF/Arch-amoAR	286	34	6.53e4-1.06e6	Li et al., 2018b
Changjiang River estuary	Arch-amoAF/Arch-amoAR	760	60	2.42e5-6.09e6	He et al., 2014
Jiulong River estuary	CrenamoA23f/CrenamoA616r	-	-	4.69e5-1.81e8	Zou et al., 2020b
San Francisco Bay estuary	Arch-amoAF/Arch-amoAR	392	67	1.40e4-3.90e7	Mosier et al., 2008
Plum Island Sound estuary	Arch-amoAF/Arch-amoAR	451	-	3.80e4-2.40e8	Bernhard et al., 2010
Bahia del Tobari estuary	Arch-amoAF/Arch-amoAR	282	42	9.17e5-5.97e6	Beman et al., 2006
Huntington Beach estuary	Arch-amoAF/Arch-amoAR	338	52	1.10e4-6.60e5	Santoro et al., 2008
Douro River estuary	Arch-amoAF/Arch-amoAR	50	23	9.00e4-8.50e5	Magalhães et al., 2009
Elkhorn Slough estuary	Arch-amoAF/Arch-amoAR	250	52	4.90e3-1.20e5	Wankel et al., 2011
Fitzroy river estuary	Arch-amoAF/Arch-amoAR	132	27	9.50e6-3.50e7	Abell et al., 2010

-: Data not mentioned in the original paper.

Table S3 Detailed community composition fractions for total archaea

Location	Thaumarchaeota	Euryarchaeota	Bathyarchaeota	Woesearchaeota	Lokiarchaeota	Thorarchaeota	Others
**Water samples**							
Liaohe River estuary	0.18	0.31	0.05	0.39	-	-	0.07
Yellow River estuary	0.40	0.48	0.02	-	-	-	0.10
Changjiang River estuary	0.48	0.46	0.03	-	-	-	0.03
Jiulong River estuary	0.28	0.39	0.29	-	-	-	0.04
Pearl River estuary	0.43	0.12	0.26	0.12	-	-	0.07
Tomoe estuary	0.35	0.26	0.39			-	-
Ashtum El Gamil estuary	0.35	0.24	0.29	-	-	-	-
Mackenzie River estuary	0.45	0.02	-	0.51	-	-	0.02
- Mississippi River estuary	0.49	0.17	0.34	-	-	-	-
Urucu estuary	0.74	0.08	0.16	-	-	-	0.02
Solimões River estuary	0.95	0.02	0.02	-	-	-	0.01
Cunha estuary	0.09	0.90	-	-	-	-	0.01
**Sediment samples**							
Liaohe River estuary	0.62	0.05	0.04	0.11	-	-	0.18
Yellow River estuary	0.28	0.42	0.04	-	-	0.15	0.11
Changjiang River estuary	0.22	0.14	0.43	0.05	-	0.07	0.09

Jiulong River estuary	0.01	0.31	0.53	-	-	-	0.15
Pearl River estuary	0.25	0.12	0.53	-	0.06	-	0.04
Orikasa River estuary	-	0.25	-	0.68	-	-	0.07
Mandovi estuary	0.02	0.58	0.04	-	-	-	0.36
Zuari estuary	0.60	0.12	0.28	-	-	-	0
Colne estuary	0.25	0.19	0.46	-	-	0.06	0.04
Severn estuary	0.14	0.04	0.55	-	-	-	0.27
Beaulieu estuary	-	0.56	0.22	0.17	-	-	0.05
Rhone River estuary	-	0.81	0.05	-	0.11	-	0.03
White Oak River estuary	-	0.13	0.70	-	-	0.09	0.08
Santos-Sao Vicente estuary	0.05	0.35	0.28	0.29	-	-	0.03

-: Data not mentioned in the original paper.

Table S4 Detailed community composition for archaeal *amoA* genes

Location	NP-α	NP-β	NP-γ	NP-δ	NP-ε	NP-η	NP-θ	NS-θ	NS-θ	NS-γ	NS-δ	NS-ε	NT-α	NT-γ	Other
**Water samples**															
Pearl River estuary	0.000	0.000	0.618	0.000	0.000	0.000	0.000	0.147	0.000	0.000	0.059	0.000	0.059	0.000	0.118
Yellow River estuary	0.000	0.000	0.643	0.000	0.000	0.094	0.000	0.053	0.000	0.000	0.164	0.000	0.000	0.023	0.023

Changjiang River estuary	0.000	0.000	0.134	0.000	0.576	0.271	0.000	0.000	0.000	0.000	0.000	0.000	0.000	0.000	0.018
Jiulong River estuary	0.000	0.000	0.970	0.000	0.020	0.000	0.000	0.000	0.000	0.000	0.000	0.000	0.000	0.000	0.010
Yong River estuary	0.000	0.000	0.238	0.095	0.048	0.381	0.000	0.000	0.000	0.000	0.238	0.000	0.000	0.000	0.000
Puget Sound estuary	0.000	0.000	0.043	0.000	0.904	0.000	0.000	0.000	0.000	0.032	0.000	0.000	0.000	0.000	0.021
**Sediment samples**															
Pearl River estuary	0.031	0.000	0.806	0.000	0.000	0.000	0.082	0.000	0.000	0.000	0.031	0.000	0.000	0.000	0.051
Yellow River estuary	0.000	0.000	0.143	0.000	0.000	0.178	0.000	0.122	0.000	0.056	0.458	0.000	0.000	0.021	0.021
Changjiang River estuary	0.355	0.019	0.102	0.373	0.064	0.053	0.000	0.000	0.000	0.000	0.026	0.000	0.000	0.000	0.008
Jiulong River estuary	0.000	0.000	0.00	0.000	0.900	0.000	0.000	0.000	0.000	0.000	0.000	0.000	0.000	0.000	0.100
San Francisco Bay estuary	0.000	0.000	0.848	0.000	0.000	0.000	0.018	0.000	0.018	0.057	0.000	0.039	0.000	0.000	0.022
Plum Island Sound estuary	0.000	0.000	0.782	0.136	0.000	0.000	0.000	0.000	0.026	0.000	0.022	0.000	0.000	0.000	0.033
Bahia del Tobari estuary	0.459	0.000	0.055	0.083	0.000	0.000	0.000	0.216	0.000	0.133	0.037	0.000	0.000	0.000	0.018
Huntington Beach estuary	0.000	0.184	0.171	0.267	0.102	0.000	0.244	0.000	0.000	0.000	0.000	0.000	0.000	0.000	0.000
Douro River estuary	0.000	0.000	0.375	0.225	0.000	0.000	0.000	0.000	0.000	0.175	0.125	0.000	0.000	0.000	0.100
Elkhorn Slough estuary	0.000	0.000	0.192	0.088	0.000	0.000	0.000	0.000	0.064	0.180	0.172	0.136	0.116	0.000	0.052
Fitzroy river estuary	0.000	0.000	0.000	0.000	0.000	0.000	0.000	0.135	0.068	0.150	0.594	0.000	0.000	0.000	0.045

Table S5 Detailed subgroups composition for *Bathyarchaeota*

Location	Bathy-17	Bathy-15	Bathy-8	Bathy-6	Bathy-14	Bathy-13	Bathy-12	Bathy-4	Bathy-3	Bathy-1	Other
Liaohe River estuary	0.101	0.492	0.193	0.181	0.000	0.000	0.000	0.000	0.000	0.000	0.034
Yellow River estuary	0.079	0.897	0.000	0.010	0.000	0.000	0.000	0.000	0.000	0.000	0.014
Changjiang River estuary	0.175	0.239	0.106	0.094	0.038	0.063	0.033	0.000	0.000	0.119	0.135
Jiulong River estuary	0.101	0.101	0.145	0.348	0.014	0.029	0.058	0.014	0.014	0.000	0.174
Pearl River estuary	0.105	0.187	0.241	0.137	0.024	0.041	0.064	0.021	0.042	0.033	0.106
Mandovi estuary	0.508	0.066	0.180	0.066	0.016	0.033	0.016	0.033	0.033	0.016	0.033
Zuari estuary	0.074	0.000	0.463	0.222	0.000	0.037	0.037	0.019	0.019	0.019	0.111
Colne estuary	0.000	0.314	0.257	0.286	0.000	0.000	0.086	0.000	0.000	0.000	0.057
Severn estuary	0.286	0.286	0.071	0.143	0.000	0.143	0.000	0.000	0.000	0.000	0.071
Beaulieu estuary	0.071	0.143	0.286	0.357	0.000	0.000	0.000	0.000	0.000	0.000	0.143
Rhone River estuary	0.063	0.031	0.250	0.094	0.000	0.000	0.000	0.000	0.000	0.500	0.063
White Oak River estuary	0.077	0.000	0.247	0.111	0.000	0.052	0.029	0.133	0.027	0.092	0.232
Santos-Sao Vicente estuary	0.053	0.307	0.040	0.200	0.000	0.013	0.013	0.000	0.000	0.347	0.027

References

Abell, G. C., Revill, A. T., Smith, C., Bissett, A. P., Volkman, J. K., & Robert, S. S. (2010). Archaeal ammonia oxidizers and nirS-type denitrifiers dominate sediment nitrifying and denitrifying populations in a subtropical macrotidal estuary. *The ISME Journal, 4*(2), 286-300.

Alves, R. J. E., Minh, B. Q., Urich, T., von Haeseler, A., & Schleper, C. (2018). Unifying the global phylogeny and environmental distribution of ammonia-oxidising archaea based on amoA genes. *Nature Communications, 9*(1), 1-17.

Baker, B. J., De Anda, V., Seitz, K. W., Dombrowski, N., Santoro, A. E., & Lloyd, K. G. (2020). Diversity, ecology and evolution of Archaea. *Nature Microbiology*, 1-14.

Beman, J. M., & Francis, C. A. (2006). Diversity of ammonia-oxidizing archaea and bacteria in the sediments of a hypernutrified subtropical estuary: Bahia del Tobari, Mexico. *Applied Environmental Microbiology 72*(12), 7767-7777.

Bernhard, A. E., Landry, Z. C., Blevins, A., José, R., Giblin, A. E., & Stahl, D. A. (2010). Abundance of ammonia-oxidizing archaea and bacteria along an estuarine salinity gradient in relation to potential nitrification rates. *Applied Environmental Microbiology, 76*(4), 1285-1289.

Elsaied, H. E., Taleb, H. T. A., Wassel, M. A., & Rashed, M. A. S. (2016). Composition of Eukaryotic and Prokaryotic Rrna Gene Phylotypes uin Guts of Adults and Fingerlings of Mugil cephalus, Inhabiting an Egyptian Mediterranean Estuary. *Journal of Phylogenetics & Evolutionary Biology, 4*(164), 2.

Galand, P. E., Lovejoy, C., Pouliot, J., Garneau, M. È., & Vincent, W. F. (2008). Microbial community diversity and heterotrophic production in a coastal Arctic

ecosystem: a stamukhi lake and its source waters. *Limnology and Oceanography, 53*(2), 813-823.

He, H., Zhen, Y., Mi, T., & Yu, Z. (2014). Community Composition and Abundance of Ammonia-Oxidizing Archaea in Sediments from the Changjiang Estuary and its Adjacent Area in the East China Sea. *Geomicrobiology Journal, 33*(5), 416–425. doi:10.1080/01490451.2014.986695

Hu, A., Hou, L., & Yu, C. P. (2015). Biogeography of planktonic and benthic archaeal communities in a subtropical eutrophic estuary of China. *Microbial Ecology, 70*(2), 322-335.

Jin, T., Zhang, T., Ye, L., Lee, O. O., Wong, Y. H., & Qian, P. Y. (2011). Diversity and quantity of ammonia-oxidizing Archaea and Bacteria in sediment of the Pearl River Estuary, China. *Applied Microbiology and Biotechnology, 90*(3), 1137–1145.

Kaku, N., Ueki, A., Ueki, K., & Watanabe, K. (2005). Methanogenesis as an important terminal electron accepting process in estuarine sediment at the mouth of Orikasa River. *Microbes and Environments, 20*(1), 41-52.

Khandeparker, L., Kuchi, N., Kale, D., & Anil, A. C. (2017). Microbial community structure of surface sediments from a tropical estuarine environment using next generation sequencing. *Ecological Indicators, 74*, 172-181.

Lazar, C. S., Biddle, J. F., Meador, T. B., Blair, N., Hinrichs, K. U., & Teske, A. P. (2015). Environmental controls on intragroup diversity of the uncultured benthic archaea of the miscellaneous C renarchaeotal group lineage naturally enriched in anoxic sediments of the White Oak River estuary (North Carolina, USA). *Environmental Microbiology, 17*(7), 2228-2238.

Li, M., Mi, T., Yu, Z., Ma, M., & Zhen, Y. (2020). Planktonic Bacterial and Archaeal Communities in an Artificially Irrigated Estuarine Wetland: Diversity, Distribution, and Responses to Environmental Parameters. *Microorganisms, 8*(2), 198.

Li, M., Wei, G., Shi, W., Sun, Z., Li, H., Wang, X., & Gao, Z. (2018). Distinct distribution patterns of ammonia-oxidizing archaea and bacteria in sediment and water column of the Yellow River estuary. *Scientific reports*, *8*(1), 1-10.

Li, Z., Jin, W., Liang, Z., Yue, Y., & Lv, J. (2013). Abundance and diversity of ammonia-oxidizing archaea in response to various habitats in Pearl River Delta of China, a subtropical maritime zone. *Journal of Environmental Sciences, 25*(6), 1195–1205.

Liu, J., Zhu, S., Liu, X., Yao, P., Ge, T., & Zhang, X. H. (2020). Spatiotemporal dynamics of the archaeal community in coastal sediments: assembly process and co-occurrence relationship. *The ISME Journal*, 1-16.

Liu, X., Li, M., Castelle, C. J., Probst, A. J., Zhou, Z., Pan, J., ... & Gu, J. D. (2018a). Insights into the ecology, evolution, and metabolism of the widespread Woesearchaeotal lineages. *Microbiome*, *6*(1), 102.

Liu, X., Pan, J., Liu, Y., Li, M., & Gu, J. D. (2018b). Diversity and distribution of Archaea in global estuarine ecosystems. *Science of the Total Environment, 637*, 349-358.

Magalhães, C. M., Machado, A., & Bordalo, A. A. (2009). Temporal variability in the abundance of ammonia-oxidizing bacteria vs. archaea in sandy sediments of the Douro River estuary, Portugal. *Aquatic Microbial Ecology, 56*(1), 13-23.

Mosier, A. C., & Francis, C. A. (2008). Relative abundance and diversity of ammonia-oxidizing archaea and bacteria in the San Francisco Bay estuary. *Environmental Microbiology, 10*(11), 3002–3016.

Santoro, A. E., Francis, C. A., de Sieyes, N. R., & Boehm, A. B. (2008). Shifts in the relative abundance of ammonia-oxidizing bacteria and archaea across physicochemical gradients in a subterranean estuary. *Environmental Microbiology, 10*(4), 1068–1079.

Singh, S. K., Verma, P., Ramaiah, N., Chandrashekar, A. A., & Shouche, Y. S. (2010). Phylogenetic diversity of archaeal 16S rRNA and ammonia monooxygenase genes from tropical estuarine sediments on the central west coast of India. *Research in Microbiology, 161*(3), 177-186.

Toyama, D., Santos-Júnior, C. D., Kishi, L. T., Oliveira, T. C. S., Garcia, J. W., Sarmento, H., ... & Henrique-Silva, F. (2017). A snapshot on prokaryotic diversity of the Solimões River basin (Amazon, Brazil). *Genetics and Molecular Research, 16*(2).

Urakawa, H., Martens-Habbena, W., Huguet, C., de la Torre, J. R., Ingalls, A. E., Devol, A. H., & Stahl, D. A. (2014). Ammonia availability shapes the seasonal distribution and activity of archaeal and bacterial ammonia oxidizers in the Puget Sound Estuary. *Limnology and Oceanography, 59*(4), 1321-1335.

Wankel, S. D., Mosier, A. C., Hansel, C. M., Paytan, A., & Francis, C. A. (2011). Spatial variability in nitrification rates and ammonia-oxidizing microbial communities in the agriculturally impacted Elkhorn Slough estuary, California. *Applied Environmental Microbiology, 77*(1), 269-280.

Webster, G., O'Sullivan, L. A., Meng, Y., Williams, A. S., Sass, A. M., Watkins, A. J., ... & Weightman, A. J. (2015). Archaeal community diversity and abundance changes along a natural salinity gradient in estuarine sediments. *FEMS Microbiology Ecology, 91*(2), 1-18.

Webster, G., Rinna, J., Roussel, E. G., Fry, J. C., Weightman, A. J., & Parkes, R. J.

(2010). Prokaryotic functional diversity in different biogeochemical depth zones in tidal sediments of the Severn Estuary, UK, revealed by stable-isotope probing. *FEMS Microbiology Ecology, 72*(2), 179-197.

Wei, G., Li, M., Li, F., Li, H., & Gao, Z. (2016). Distinct distribution patterns of prokaryotes between sediment and water in the Yellow River estuary. *Applied Microbiology and Biotechnology, 100*(22), 9683-9697.

Xie, W., Luo, H., Murugapiran, S. K., Dodsworth, J. A., Chen, S., Sun, Y., ... & Zhang, C. L. (2018). Localized high abundance of Marine Group II archaea in the subtropical Pearl River Estuary: implications for their niche adaptation. E*nvironmental Microbiology, 20*(2), 734-754.

Zhang, Q., Tang, F., Zhou, Y., Xu, J., Chen, H., Wang, M., & Laanbroek, H. J. (2015). Shifts in the pelagic ammonia-oxidizing microbial communities along the eutrophic estuary of Yong River in Ningbo City, China. *Frontiers in Microbiology, 6*, 1180.

Zhang, Y., Xie, X., Jiao, N., Hsiao, S. Y., & Kao, S. J. (2014). Diversity and distribution of amoA-type nitrifying and nirS-type denitrifying microbial communities in the Yangtze River estuary. *Biogeosciences, 11*(8), 2131.

Zhou, Z., Pan, J., Wang, F., Gu, J. D., & Li, M. (2018). Bathyarchaeota: globally distributed metabolic generalists in anoxic environments. *FEMS Microbiology Reviews, 42*(5), 639-655.

Zhou, Z., Liu, Y., Lloyd, K. G., Pan, J., Yang, Y., Gu, J. D., & Li, M. (2019). Genomic and transcriptomic insights into the ecology and metabolism of benthic archaeal cosmopolitan, Thermoprofundales (MBG-D archaea). *The ISME journal*, *13*(4), 885-901.

Zhou, Z., Liu, Y., Xu, W., Pan, J., Luo, Z. H., & Li, M. (2020). Genome-and

community-level interaction insights into carbon utilization and element cycling functions of Hydrothermarchaeota in hydrothermal sediment. *Msystems*, *5*(1).

Zou, D., Pan, J., Liu, Z., Zhang, C., Liu, H., & Li, M. (2020a). The distribution of Bathyarchaeota in surface sediments of the Pearl River estuary along salinity gradient. *Frontiers in Microbiology*, *11*, 285.

Zou, D., Wan, R., Han, L., Xu, M. N., Liu, Y., Liu, H., ... & Li, M. (2020b). Genomic Characteristics of a Novel Species of Ammonia-oxidizing Archaea from the Jiulong River Estuary. *Applied and Environmental Microbiology*. doi: 10.1128/AEM.00736-20
